# Adsorption characteristics and mechanism of p-nitrophenol by pine sawdust biochar samples produced at different pyrolysis temperatures

**DOI:** 10.1038/s41598-020-62059-y

**Published:** 2020-03-20

**Authors:** Lanqi Liu, Guozhi Deng, Xianyang Shi

**Affiliations:** School of Resources and Environmental Engineering, Anhui University, Hefei, 230601 China

**Keywords:** Environmental sciences, Environmental chemistry

## Abstract

Biochar is becoming a low-cost substitute of activated carbon for the removal of multiple contaminants. In this study, five biochar samples derived from pine sawdust were produced at different pyrolysis temperatures (300 °C–700 °C) and used adsorbents to remove p-nitrophenol from water. Results indicate that, as the pyrolysis temperature increases, the surface structure of biochar grows in complexity, biochar’s aromaticity and number of functional group decrease, and this material’s polarity increases. Biochar’s physiochemical characteristics and dosage, as well as solution’s pH and environmental temperature significantly influence the p-nitrophenol adsorption behavior of biochar. p-nitrophenol adsorption onto biochar proved to be an endothermic and spontaneous process; furthermore, a greater energy exchange was observed to take place when biochar samples prepared at high temperatures were utilized. The adsorption mechanism includes physical adsorption and chemisorption, whereas its rate is mainly affected by intra-particle diffusion. Notably, in biochar samples prepared at low temperature, adsorption is mainly driven by electrostatic interactions, whereas, in their high-temperature counterparts, p-nitrophenol adsorption is driven also by hydrogen bonding and π–π interactions involving functional groups on the biochar surface.

## Introduction

p-nitrophenol, a common pollutant found in water and wastewater, is widely used in the manufacture of petrochemicals, medicines, dyes, and insectifuges, as well as in leather coloring^1^. Once it enters the bloodstream, p-nitrophenol can convert hemoglobin into methemoglobin, which may in turn cause liver damage, anemia, dyspnea and other related symptoms^2^. As a result of its persistency, bioaccumulation, and toxicity, p-nitrophenol has been defined as a priority pollutant by the United States Environmental Protection Agency^3^. Therefore, the effective removal of p-nitrophenol from aqueous media, or the reduction of its concentration, has attracted a great deal of research attention.

Many treatment techniques have been employed to reduce the environmental impact of p-nitrophenol, including biodegradation^4^, catalytic reduction^5^, liquid membrane separation^6^, and adsorption^7^. Among these techniques, adsorption is the most convenient because of its operating flexibility and design simplicity, as well as the ease of regeneration of the raw materials on which it relies^8^. However, the high cost of adsorbents limited this technique’s large-scale application. Developing cheap and efficient p-nitrophenol adsorbent materials is thus a desirable research and practical objective.

Biochar is regarded as a typically cheap and effective adsorbent, because of its microporous structure and abundant active surface^9,10^. The adsorption capacity of biochar is usually determined by its physicochemical properties, which vary with the properties of the raw materials used to produce it, the temperature of pyrolysis, and other preparation parameters^11^. In particular, the pyrolysis temperature plays a crucial role in the performance of biochar, as its value influences characteristics such as elemental composition, identity and number of functional groups, specific surface area, and aromaticity level^11,12^.

As a by-product of sawmill activity, sawdust is often combusted directly to generate heat, but this process causes the release of large amounts of carbon dioxide, an environmental pollutant. Therefore, the conversion of sawdust into oil and/or biochar as an environmentally friendly alternative to combustion is attracting increasing interest. Several types of sawdust have been transformed into biochar, and the pollutant adsorption behavior of the various materials thus obtained has been reported. The adsorption capacity of sawdust-derived biochar is affected mainly by the type of sawdust utilized to produce it, the pyrolysis temperature and time, the identities of the pollutants, and the environmental conditions in which the adsorption takes place^13,14^. For the large-scale application of biochar to environmental p-nitrophenol adsorption, identifying a cheap type of biomass that can be converted by a low-cost procedure to biochar characterized by high adsorption capacity is a key objective. However, little information is currently available on the influence that pyrolysis temperature has on the adsorption behavior of sawdust-derived biochar toward p-nitrophenol and on the mechanism of the relevant process.

In the present study, waste pine sawdust was used as raw material for the preparation of biochar at five different pyrolysis temperatures. The effects that biochar properties and environmental factors have on the adsorption of p-nitrophenol by the biochar were investigated. The adsorption mechanisms were also explored using kinetics, isotherms, and thermodynamics. The results of this study may provide theoretical guidance in the preparation of pine sawdust biochar and the use of the biochar samples thus obtained for the removal of p-nitrophenol from aqueous media.

## Materials and Methods

### Biochar production

Pine sawdust was rinsed with deionized water and dried in an oven at 90 °C for 24 h^15^. This material was heated to a preset temperature (300 °C, 400 °C, 500 °C, 600 °C, or 700 °C) implementing a program wherein the rate of temperature growth was 7 °C/min. The system was then kept at the desired temperature for 1 h under a suitable nitrogen flow rate (有没有具体的流速) in a furnace. The furnace was then allowed to cool to room temperature. The biochar samples obtained were then labeled PC300, PC400, PC500, PC600, and PC700, where each suffix number indicates the pyrolysis temperature employed to produce each sample. The milled biochar samples were thoroughly rinsed to remove ash, then dried, and finally screen-sieved to obtain 16–80 mesh-size particles.

### Biochar characterization

The carbon (C), hydrogen (H), and nitrogen (N) contents of each biochar sample were determined using an Element Analyzer (Elementar Vario EL III, Frankfurt, Germany). The yield data of biochar were calculated based on mass balance. The ash was analyzed according to the approach reported by He *et al*.^16^ and its weight percent calculated using the following equation:

1 $$Ash( \% )=\frac{{W}_{f}}{{W}_{i}}\times 100$$

where *W*_*f*_ and *W*_*i*_ represent the final and initial mass of the biochar sample, respectively. The biochar samples’ oxygen (O) content was calculated on the basis of the mass balance assuming that whatever was not ash, C, H, and N had to be O^17^:

2 $$O \% =100 \% -Ash \% -C \% -H \% -N \% $$

The surface area and pore properties of biochar were analyzed using a surface area and pore size analyzer (Micrometrics ASAP 2460, Norcross, USA). The surface structure and morphology of all biochar samples were observed with a FIELD Scanning Electron Microscope (SEM, Hitachi S-4800, Tokyo, Japan). The pH_pzc_ (pH at point of zero charges) values of biochar samples were measured using a Zeta potential analyzer (Nano Brook Zeta Plus, Suffolk, USA). Fourier-transform infrared (FTIR) spectra (recorded using a Bruker Vertex 80 spectrometer, Karlsruhe, Germany) were collected in the 400–4000 cm^−1^ wavenumber range to identify the functional groups present on the surface of biochar samples.

### Adsorption experiments

A stock 2 g/L p-nitrophenol solution was prepared with deionized water. Aliquots of this solution were diluted to reach the required concentrations before adsorption experiments were conducted. All adsorption experiments were performed in duplicate in 200 mL conical flasks at 35 °C while shaking at a speed of 135 rpm for 4 h. The solid–liquid concentration of the p-nitrophenol solution (20 mL, 100 mg/L) and biochar was made to vary from 1 to 9 g/L, in order to examine the influence that biochar dosage had on p-nitrophenol adsorption. To evaluate the effect of pH (3–11) on p-nitrophenol adsorption by biochar, through experiments whereby 0.1 g biochar samples were added into 20 mL p-nitrophenol solutions in 200 mL conical flasks, the pH of the 100 mg/L p-nitrophenol solution was adjusted to 3, 4, 5, 6, 7, 8, 9, 10, or 11 using 0.1 mol/L NaOH or 0.1 mol/L HCl aqueous solutions. Moreover, to evaluate the influence of ionic strength on p-nitrophenol adsorption by biochar, the NaCl concentration in the 100 mg/L p-nitrophenol solution at pH 6 was made to range from 0 to 0.8 mol/L. To investigate the adsorption isotherm and kinetics of biochar-driven p-nitrophenol removal, the initial mass concentration gradients of p-nitrophenol solution were made to vary from 50 to 800 mg/L—the solution’s pH was 6, the amount of added biochar was 0.1 g, and the contact time between biochar and the p-nitrophenol solution was 48 h. Notably, at appropriate time intervals, the reaction mixtures were collected and centrifuged for 10 min at 12,000 rpm. The concentration of p-nitrophenol in the supernatants thus obtained was then determined recording the solution’s absorbance at 317 nm with an UV-vis spectrophotometer (Shimadzu, UV-2550, Tokyo, Japan).

### Data analysis

The biochar sample’s adsorption capacity at time t is calculated by the following equation^18^:

3 $${q}_{t}=\frac{({C}_{0}-{C}_{t})\cdot V}{W}$$

where *C*_*0*_ and *C*_*t*_ (mg/L) indicate the concentration of p-nitrophenol during the adsorption experiment at the initial time and at time t, respectively. V represents the volume (in L) of the p-nitrophenol solution, and W represents the mass (in g) of the biochar sample utilized in the experiment.

The pseudo-first-order, pseudo-second-order, and Elovich kinetic models as well as the intra-particle diffusion model were used to investigate the kinetics of p-nitrophenol adsorption by biochar^19^. The equations defining these models are reported in the section of the text that follows^20^.

Pseudo-first-order kinetic model:

4 $${q}_{t}={q}_{e}(1-{e}^{-{k}_{1}t})$$

Pseudo-second-order kinetic model:

5 $${q}_{t}=\frac{{k}_{2}{q}_{e}^{2}t}{1+{k}_{2}{q}_{e}t}$$

Elovich kinetic model:

6 $${q}_{t}=\left(\frac{1}{b}\right)\mathrm{ln}\,ab+\left(\frac{1}{b}\right)\mathrm{ln}\,t$$

Intra-particle diffusion model:

7 $${q}_{t}={k}_{i}{t}^{1/2}+{C}_{i}$$

where *q*_*t*_ (mg/g) and q_e_ (mg/g) represent the amount of p-nitrophenol adsorbed at time t and at equilibrium, respectively; a (mg/g·min) and b (g/mg) represent the initial adsorption rate and the surface coverage constant, respectively; k_1_ (1/min), k_2_ (g/mg·min), and k_i_ (mg·min^1/2^/g) are the rate constants for pseudo-first-order, pseudo-second-order, and intra-particle diffusion models; C_i_ (mg/g) is a parameter related to the thickness of the boundary layer.

Langmuir, Freundlich, and Temkin isotherm models were used to evaluate the adsorption behavior of biochar with respect to p-nitrophenol. The equations defining these three models are reported in the section of the text that follows.

Langmuir model^21^:

8 $${{q}}_{{e}}{=}\frac{{{q}}_{{m}}{{K}}_{{L}}{{C}}_{{e}}}{{1}{+}{{K}}_{{L}}{{C}}_{{e}}}$$

Freundlich model:

9 $${{q}}_{{e}}{=}{{K}}_{F}{{C}}_{{e}}^{\frac{{1}}{{n}}}$$

Temkin model:

10 $${q}_{e}=\frac{RT}{{b}_{T}}\,\mathrm{ln}({K}_{T}{C}_{e})$$

where q_m_ (mg/g) is the assumed maximum adsorption capacity, C_e_ (mg/L) is the residual p-nitrophenol concentration at adsorption equilibrium, n is a parameter measuring the surface inhomogeneity of adsorbents, R is the universal gas constant (8.314 J/mol), T (K) is the absolute temperature, and K_L_ (L/g), K_F_ (mg^1−1/n^·L^1/n^/g), K_T_ (L/g), and b_T_ are the Langmuir, Freundlich, and Temkin parameters, respectively.

## Results and discussion

### Characterization of biochar

The characteristics of biochar depend on the pyrolysis temperature at which the material is prepared and so does biochar’s adsorption capacity. Elemental analysis results (Table 1) indicated that in the 300–700 °C rage, the C content of biochar increased alongside the pyrolysis temperature. This observation descends from the fact that aromatic polymerization and graphene nucleation occurred on the cellulose and hemicellulose of pine sawdust^8^. The H and O content in biochar decreased as a result of the dehydration and dehydrogenation of biochar during the heating process. As the pyrolysis temperature increased, the values for the H/C and (O + N)/C ratios decreased, as a consequence, PC700 is expected to be characterized by the highest aromaticity and the lowest polarity among all the prepared biochar samples^22,23^.

**Table 1:** Physicochemical properties of the biochar samples obtained at different pyrolysis temperatures.

Sample	PC300	PC400	PC500	PC600	PC700
C%	19.07	27.67	46.93	52.13	56.96
H%	4.37	3.83	3.27	3.08	2.89
N%	0.14	0.18	0.21	0.23	0.26
O%	74.17	64.56	44.95	39.81	34.91
Ash%	2.25	3.76	4.64	4.75	4.98
H/C atomic ratio	0.23	0.14	0.07	0.06	0.05
(O + N)/C atomic ratio	3.90	2.34	0.96	0.77	0.62
yield (wt%)	91.08	80.26	76.92	73.84	71.33
pH_pzc_	3.12	4.84	5.29	5.83	5.94
N_2_-BET area (m^2^/g)	21.583	47.745	331.291	432.737	397.864
Pore volume (cm^3^/g)	0.021	0.028	0.182	0.233	0.227
Pore size (nm)	3.873	2.375	2.182	2.117	2.249

As can be evinced from the data in Table 1, an increase in pyrolysis temperature is associated with a decrease in biochar yield (91.8%–71.33%). When pyrolysis was carried out at a temperature of 500 °C, the yield of biochar decreased to 76.92% (PC500) from a value of 91.08% measured when pyrolysis was performed at 300 °C (PC300). The observed reduction in biochar yield may be due to rapid biomass vaporization, condensation, and carbonization. In comparison with the case of the biochar produced at a lower pyrolysis temperature, the yield of biochar prepared at a higher pyrolysis temperature (500–700 °C) decreases more indistinctively, which implies that the pyrolysis temperature rarely affects the yield of highly carbonized biochar. Therefore, it can be inferred that 500 °C is the temperature at which the physical properties of pine sawdust biochar undergo a significant change^16^. Notably, pine sawdust biochar production is still associated with a large yield when pyrolysis is performed at higher temperatures; interestingly, such temperatures are higher than those reported earlier for the pyrolysis of biomass samples^24,25^.”

**Figure 1:**
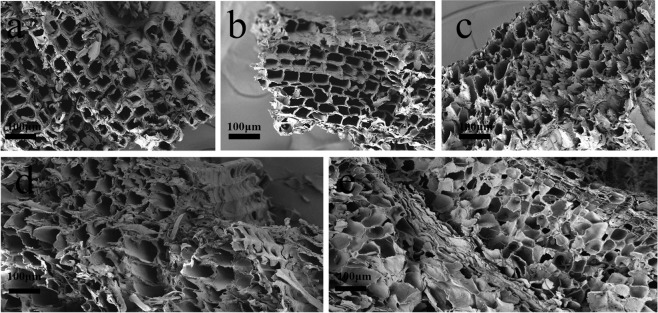
Surface morphology of five biochar samples obtained at different pyrolysis temperatures as made evident by scanning electron microscopy images: (**a**) PC300, (**b**) PC400, (c) PC500, (**d**) PC600, and (**e**) PC700.

The values for the surface area and pore properties (Table 1) of the biochar samples indicate that, as the pyrolysis temperature increases, the structural characteristics of the samples thus obtained improve in terms of their ability to act as adsorbents. A significant increase in the values for the surface area and pore volume was observed on going from PC300 (21.583 m^2^/g and 0.021 cm^3^/g, respectively) to PC600 (432.737 m^2^/g and 0.233 cm^3^/g, respectively), suggesting that the formation of biochar’s fibrous tubular structure was brought about by the release of volatile components, a process that becomes more effective as the pyrolysis temperature increases^26^. Interestingly, when the pyrolysis temperature was 700 °C, the relevant biochar sample (PC700) displayed a slight decrease in surface area (397.864 m^2^/g) and pore volume (0.227 cm^3^/g) with respect to PC600. This observation could be the result of structural damage and pore blockage experienced by the biochar as a consequence of an excessively high pyrolysis temperature^27,28^. These results were also confirmed by the microscopic features of the materials. SEM images of the surfaces of the various samples (Fig. 1) show that the biochar samples produced at 300 °C and 400 °C are characterized by the presence of regularly shaped and interspersed tubular structures. As the pyrolysis temperature increased, however, these tubular structures underwent deformation and the surface roughness of biochar increased. In particular, the surface structure became looser, which in turn increased the biochar’s surface roughness. When the pyrolysis temperature reached the value of 700 °C, the obtained sample’s tubular structures collapsed inwards to give rise to a layered structure, resulting in the blockage of micropores.

### Factors that impact p-nitrophenol adsorption

#### Effect of adsorbent dosage on p-nitrophenol adsorption

The extent of p-nitrophenol adsorption by PC300 was plotted against PC300 dosage (ranging between 1 and 16 g/L) as a typical example (Supplementary Fig. S1). The adsorbent showed good adsorption capacity at low dose. The amount of p-nitrophenol adsorbed decreased and the removal rate increased as the biochar dosage increased. Most biochar samples were characterized by a high removal rate at a dosage of 5 g/L; and the p-nitrophenol removal rate were as follows: 38.29%, 77.06%, 96.93%, 98.85%, and 99.61%, for PC300, PC400, PC500, PC600, and PC700, respectively. Notably, when the adsorbent dosage grew above a threshold of 10 g/L, a further increase in adsorbent dosage was associated with an almost unchanged p-nitrophenol removal rate, although the adsorption amount kept decreasing. This trend may due to the fact that, as the biochar dosage increases, the degree of freedom of p-nitrophenol becomes limited, and the active sites in biochar become saturated and undergo aggregation, thereby reducing the material’s adsorption capacity^29^. Considering the relevant p-nitrophenol removal rate, the biochar utilization rate, and the cost, a biochar dosage of 5 g/L was selected to conduct the subsequent experiments.

#### Effect of the solution’s pH on p-nitrophenol adsorption

**Figure 2:**
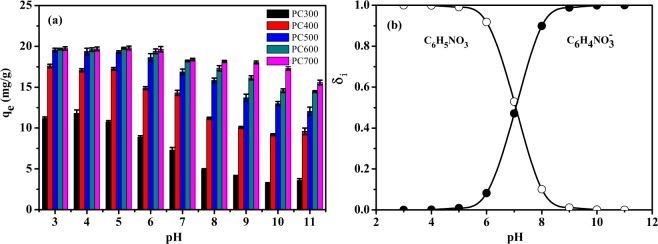
Effect of the solution’s pH on p-nitrophenol adsorption by biochar. (**a**) Adsorption amount of p-nitrophenol at different pH values. Notably, the adsorption reaction was conducted at a 100 mg/L concentration of p-nitrophenol, 0.1 g/0.02 L adsorbent dosage, and 35 °C temperature. The solution’s pH was adjusted to 3–11 by adding 0.1 mol/L NaOH or 0.1 mol/L HCl, and the pH value was basically kept constant during the adsorption process. Data comprise the average of duplicate experiments, and error bars indicate the standard error. (**b**) Species distribution of p-nitrophenol at different pH values.

As can be evinced from the data reported in Fig. 2a, at pH <5, the pH of the aqueous solution had little effect on the p-nitrophenol adsorption by biochar. This observation stems from the fact that p-nitrophenol (P_Ka_ = 7.15) is mainly present as a neutral species (C_6_H_5_NO_3_) in the mentioned pH range (Fig. 2b), so that no strong electrostatic repulsion exists between biochar and p-nitrophenol, which would affect adsorption^30^. By contrast, biochar’s p-nitrophenol adsorption capacity evidently decreases as the pH of the solution increases from 6 to 11, suggesting that the pH_pzc_ values of the five biochar samples are lower than 6 (Table 1), so that the surfaces of these samples are negatively charged as a consequence of the ionization of the functional groups present on the said surfaces. Importantly, p-nitrophenol exists mainly in anionic form (C_6_H_4_NO_3_^−^) at pH values above 7, so that a strong electrostatic repulsion exists between biochar and p-nitrophenol. Notably, however, despite this electrostatic repulsion, PC500, PC600, and PC700 still displayed a strong p-nitrophenol adsorption capacity even when the pH value increased. This observation indicates that biochar samples PC500, PC600, and PC700 interact strongly with p-nitrophenol via H bonding and π–π interactions.

#### Effect of ionic strength on p-nitrophenol adsorption

The influence of ionic strength on the adsorption of p-nitrophenol by various biochar samples was examined. The results of the relevant experiments are reported in Supplementary Fig. S2. Notably, as the NaCl concentration increased, the q_e_ value initially increased to then decrease or decreased first to then increase; moreover, as the concentration of NaCl varied, the amount of p-nitrophenol adsorbed on biochar fluctuated within a small range. This trend may be the result of a balance between two opposite effects in solution. On the one hand, the solubility of non-electrolytes decreases as the solution’s ionic strength increases. Therefore, increases in the solution’s electrolyte concentration cause an increase in biochar’s adsorption capacity resulting from a decrease in p-nitrophenol solubility^30,31^. On the other hand, above a certain value of the electrolyte concentration, ions present in solution will compete with p-nitrophenol for the adsorption onto biochar, leading to a decrease in the amount of p-nitrophenol adsorbed^32^.

### Analysis of the adsorption process

#### Adsorption kinetics

**Figure 3:**
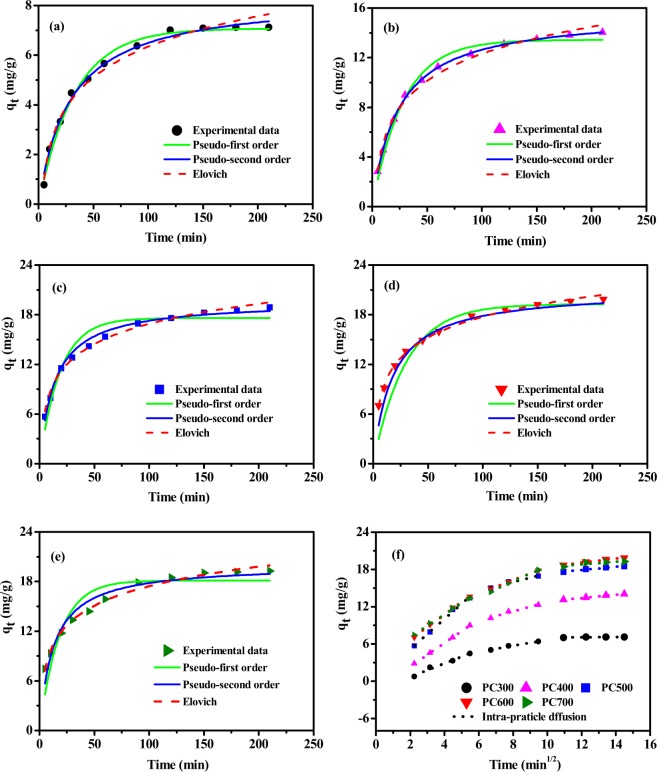
Use of various models to fit the kinetic data recorded for the adsorption of p-nitrophenol onto biochar samples (q_t_: amount of p-nitrophenol adsorbed at time point t) prepared at different pyrolysis temperatures: (**a**) PC300, (**b**) PC400, (**c**) PC500, (**d**) PC600, and (**e**) PC700 via the nonlinear regression method; (**f**) intra-particle diffusion model for five biochar samples. Please note that in the mentioned adsorption reaction, the concentration of p-nitrophenol was 100 mg/L and the dosage of biochar was 0.1 g/0.02 L. The adsorption experiments were conducted at a pH of 6 and at 35 °C, and the amount of p-nitrophenol adsorbed onto biochar was measured at the indicated time points.

The results of kinetics experiments were used to infer the reaction type of the p-nitrophenol adsorption process as well as the p-nitrophenol removal rate and the mechanism of diffusion of p-nitrophenol into biochar. The adsorption kinetic data were fitted using pseudo-first-order, pseudo-second-order, and Elovich models to explore the possible mechanism of p-nitrophenol adsorption on biochar samples prepared at different pyrolysis temperatures (Fig. 3 and Table 2). Results indicate that three models can satisfactorily fit the kinetic data obtained for biochar samples produced at low pyrolysis temperature (PC300 and PC400), based on the high R^2^ (0.982–0.999) and low SSE (0.098–0.460) values. In particular, fitting the kinetic data with the pseudo-second-order kinetic model was associated with the largest R^2^ (>0.99) and the smallest SSE (0.098–0.195) values, and the adsorption capacity (q_e_) values obtained from the pseudo-second-order model are close to the experimental values (q*). On the basis of the assumptions of this model, the adsorption rate of p-nitrophenol onto “low-temperature” biochar samples is affected by the surface active sites present in the adsorbents, and the adsorption process is controlled by chemisorption. On the other hand, the Elovich model allowed a better fit of the kinetic data recorded for biochar samples obtained at high pyrolysis temperatures (PC500, PC600, and PC700), as made evident by the relatively high R^2^ (0.989–0.995) and low SSE (0.276–0.689) values and more approximate q_e_. According to the hypothesis at the basis of this model, the energydistribution on the surface of the adsorbent was uneven and chemical adsorption occurred^20,33^.

**Table 2:** Values for the constants of the kinetic models used to fit the experimental data on the adsorption of p-nitrophenol on biochar samples prepared at different pyrolysis temperatures.

Sample	PC300	PC400	PC500	PC600	PC700
q* (experimental value)	7.1181	14.0297	18.491	19.8862	19.2583
**Pseudo-first order**					
q	7.6715	13.4334	17.6011	19.2739	18.1302
k_1_	0.0303	0.0354	0.0531	0.0337	0.0547
SSE	0.2649	0.4601	0.7888	1.8754	1.4942
R^2^	0.9824	0.9828	0.9603	0.8454	0.8449
**Pseudo-second order**					
q_e_	7.0871	14.2018	18.6552	21.0357	20.0589
k_2_	0.0041	0.0027	0.0037	0.0027	0.0039
SSE	0.1945	0.0983	0.1776	0.9609	0.7946
R^2^	0.9904	0.9992	0.9679	0.9431	0.9562
**Elovich**					
q_e_	7.1748	14.4387	19.4924	20.3989	19.9607
a	0.6282	1.5846	4.2774	5.0375	5.7226
b	0.5627	0.3186	0.2844	0.2788	0.2939
SSE	0.2025	0.3828	0.6887	0.2762	0.4002
R^2^	0.9896	0.9881	0.9897	0.9952	0.9889
**Intra-particle diffusion**					
k_i1_	1.099	1.9063	2.4338	2.022	1.8304
C_i1_	0.2229	0.461	1.3107	2.6471	3.4365
R^2^	0.9804	0.9999	0.993	0.9961	0.9966
k_i2_	0.4752	0.7414	0.9858	1.0598	1.2466
C_i2_	1.897	3.3176	5.5066	7.8012	6.1088
R^2^	0.9765	0.9533	0.9325	0.9994	0.9931
k_i3_	0.031	0.2679	0.2521	0.3341	0.2151
C_i3_	6.6865	10.1778	14.8832	15.084	16.2268
R^2^	0.9756	0.9908	0.9641	0.9878	0.9775

The limited step of the diffusion rate of p-nitrophenol onto biochar was further investigated using the intra-particle diffusion model. The adsorption process can be divided into three different stages: (1) liquid film diffusion drives the transfer of p-nitrophenol from the solution to the surface of biochar (5–30 min), and biochar’s adsorption sites are abundant and readily interact with p-nitrophenol; (2) as the remaining empty adsorption sites and the solute concentration decrease, the intra-particle diffusion progressively slows down (45–90 min); and (3) as the adsorption sites are completely saturated, the adsorption reaction reaches a state of equilibrium (120–210 min). As can be evinced from the values of the calculated parameters reported in Table 2, the R^2^ value (> 0.93) of the second line segment is higher than its counterparts of the second and the third line segments, indicating that intra-particle diffusion is the main mechanism of p-nitrophenol adsorption. Evidence suggests that, as the pyrolysis temperature increased, so did the particle diffusion rate constant (k_i_) during the second stage of the adsorption process, indicating that increases in the value of the temperature at which the biochar samples are obtained cause an increase in the number of pores located on the biochar’s surface. All C_i_ parameters were non-zero, indicating that the adsorption rate of p-nitrophenol on biochar was not only limited by diffusion^34^.

#### Adsorption isotherms

**Figure 4:**
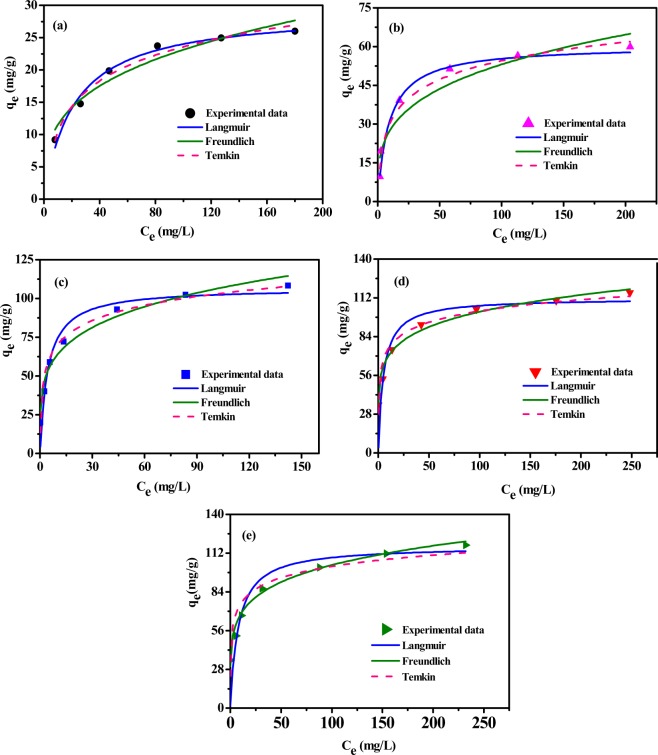
Isotherm models used to fit experimental data for the p-nitrophenol adsorption on biochar samples obtained at different pyrolysis temperatures via the nonlinear regression method: (**a**) PC300, (**b**) PC400, (**c**) PC500, (**d**) PC600, and (**e**) PC700.

Adsorption isotherms can provide information on the interactions between adsorbate and adsorbent, which is helpful when trying to optimize the use of adsorbent^35^. Langmuir, Freundlich, and Temkin isotherm models were chosen to evaluate the p-nitrophenol adsorption process (Fig. 4). The Langmuir isotherm model shows that the active sites on the surface of the adsorbents were evenly distributed and that monolayer adsorption took place on the said surface. The Freundlich isotherm model assumes that adsorption occurs on a heterogeneous surface^7,36^. According to the Temkin isotherm model, finally, a linear inverse relationship exists between adsorption heat and coverage, and binding energies are distributed homogeneously^37^.

For the adsorption experiments, a stock p-nitrophenol solution (2 g/L) was used to prepare solutions characterized by a range of p-nitrophenol concentrations (50, 100, 200, 300, 400, 500, 600, 700, and 800 mg/L); each solution was separately added to an airtight conical bottle containing 0.1 g of biochar. The reaction mixture-containing conical bottles were kept in an electrically thermostatic reciprocating shaker set to a rate of 135 rpm at 35 °C for 48 h to reach adsorption equilibrium

The fitted parameters reported in Table 3 indicated that, in the cases of the biochar samples PC300 and PC400, both the Langmuir and Temkin models display high R^2^ values. The maximum adsorption quantity (q_max_) of p-nitrophenol fitted by the Langmuir model increased alongside the pyrolysis temperature. In particular, PC600 (111.713 mg/g) and PC700 (117.165 mg/g) displayed the highest adsorption capacity, which is in good agreement with the development of the surface structures of pine sawdust biochar. Compared with the case of the Langmuir model, fitting the experimental data collected for PC300 and PC400 with the Temkin model was associated with lower SSE (0.561–1.532) and higher R^2^ (>0.98) values, indicating that the latter is a more suitable model to explain the adsorption behavior of the mentioned biochar samples. In addition, fitting the experimental kinetic data with the Freundlich model was associated with the maximum R^2^ (0.968–0.997) and the minimum SSE (2.103–4.290) values, as well as a high value for the n parameter (≥4.529), for PC500, PC600, and PC700, indicating that the adsorption of p-nitrophenol on biochar samples prepared at high pyrolysis temperatures mainly occurs on heterogeneous surfaces. This conclusion is consistent with the results of kinetic studies.

**Table 3:** Values for the constants of the isothermal models used to fit the experimental data on the adsorption of p-nitrophenol on biochar samples prepared at different pyrolysis temperatures.

Sample		PC300	PC400	PC500	PC600	PC700
Langmuir	K_L_	0.047	0.109	0.226	0.196	0.129
	Q_m_	29.166	60.348	106.789	111.713	117.165
	R^2^	0.981	0.963	0.957	0.946	0.946
	SSE	0.622	2.724	3.687	5.163	5.320
Freundlich	K_F_	5.711	14.362	38.341	47.985	44.509
	n	3.292	3.523	4.529	6.116	5.466
	R^2^	0.935	0.944	0.968	0.995	0.997
	SSE	1.033	3.024	4.290	2.103	2.776
Temkin	b_T_	446.028	245.158	180.739	217.648	217.489
	K_T_	0.606	1.845	14.135	60.482	59.738
	R^2^	0.988	0.986	0.958	0.944	0.873
	SSE	0.561	1.532	3.034	5.135	7.787

#### Thermodynamic analyses

In order to further investigate the sorption mechanisms, a thermodynamic analysis was conducted on the data obtained for the adsorption of p-nitrophenol onto the five biochar samples at three different temperatures (Supplementary Fig. S3). At the same equilibrium concentrations, the p-nitrophenol adsorption capacity of biochar increased as the temperature of the experiment increased, indicating that the said increase in the experimental temperature was beneficial to the adsorption process. The slope and intercept of the straight line obtained plotting lnK versus 1/T represent the values of ΔH and ΔS of the adsorption reaction (Table 4), respectively^38^. The fact that ΔH is positive and that its value increases as the pyrolysis temperature increases indicates that the adsorption process is endothermic and that p-nitrophenol has more energy when adsorbed onto high-temperature biochar samples than when adsorbed onto low-temperature samples. This result indicates that the interaction between p-nitrophenol and the surface active sites of high-temperature biochar samples is stronger than that between p-nitrophenol and the surface active sites of low-temperature biochar samples, which may be one of the reasons explaining why PC700 displayed the strongest adsorption capacity among the prepared biochar samples^23^. The negative value of ΔG means that the adsorption of p-nitrophenol onto biochar is a spontaneous process, and the absolute number of it is less than 40, indicating that physical adsorption occurs^2^. In addition, the positive value of ΔS reflects the fact that the increasing in randomicity of the interface between p-nitrophenol and biochar in the adsorption process.

**Table 4:** Thermodynamic parameters for the adsorption of p-nitrophenol onto biochar samples obtained at different pyrolysis temperatures.

Sample	lnK	T (K)	ΔG (kJ/mol)	ΔH (kJ/mol)	ΔS (J/(mol∙K))
PC300	1.5240	288	−3.6491	13.068	49.908
	1.7410	298	−4.3136		
	1.8776	308	−4.8081		
PC400	2.2470	288	−5.3803	17.309	78.503
	2.3841	298	−5.9071		
	2.7187	308	−6.9622		
PC500	2.7548	288	−6.5964	25.747	112.913
	3.3407	298	−8.2768		
	3.4480	308	−8.8294		
PC600	1.8776	288	−4.4959	37.218	144.799
	2.3841	298	−5.9071		
	2.8873	308	−7.3936		
PC700	2.1546	288	−5.1591	42.337	164.577
	2.6225	298	−6.4977		
	3.3056	308	−8.4647		

#### Adsorption mechanism

To verify the adsorption mechanisms, the FTIR spectra of five biochar samples and the corresponding samples of biochar loaded with p-nitrophenol (i.e., biochar samples examined after being used in p-nitrophenol adsorption experiments) were collected. In Supplementary Fig. S4 are reported spectral data providing information on the influence that the pyrolysis temperature and p-nitrophenol adsorption exhibited on the identity and abundance of functional groups present on the surface of biochar. These data indicate that PC300 and PC400 retained a high number of such functional groups, confirming the results of elemental analyses. However, as the pyrolysis temperature increased to 500 °C, the peak at 3,400 cm^−1^ (due to the stretching vibration of the –OH bond) and those at 2,925 cm^−1^ and 2,860 cm^−1^ (due to the stretching vibration of alkane C–H bonds) decreased significantly in intensity, indicating that cellulose and lignin present in the biochar had undergone dehydration between 400 °C and 500 °C^39^. Notably, the peaks at 1,707 and 1,450–1,600 cm^−1^ are assigned to the stretching vibrations of aromatic C=O and C=C bonds, respectively. In comparison with the spectra of biochar, the spectra of the biochar loaded with p-nitrophenol (i.e., biochar after having adsorbed p-nitrophenol) were characterized by shifts in the positions and intensities of the bands, indicating that many interactions are established during the p-nitrophenol adsorption process. The peaks appearing between 1,090 and 1,250 cm^−1^ are assigned to C–O and C–O–C stretching vibrations^40^ of alcohols, phenols, and ether or ester groups. Based on the decrease in intensity of the C–O–C stretching vibrations observed in the case of biochar loaded with p-nitrophenol (with respect to “pure” biochar), and the shift of the position of the peak due to –OH from 3,400 cm^−1^, in the case of biochar, to 3,464 cm^−1^, in the case of biochar loaded with p-nitrophenol, the obvious conclusion can be drawn that hydrogen bonding interactions exist between p-nitrophenol and biochar^41^. The C=C and C=O stretching vibrations observed at 1,450 cm^−1^ in the case of biochar shifted to 1,586 cm^−1^ after p-nitrophenol adsorption, indicating that the C=C and C=O groups are involved in the adsorption process, which means that the π-electron region of the aromatic structures of biochar interacts with the electron acceptor on the aromatic ring of p-nitrophenol *via* π–π interactions^42^. In addition, π − π interactions are often more intense in small biochar pores than in large ones, which is consistent with our observation that biochar samples were obtained at a high temperature, which were found to comprise pores of smaller size, and display superior adsorption capacity to their counterparts obtained at low temperature.

## Conclusions

The practicability of using recycled pine sawdust to produce biochar that can be employed as an adsorbent in the adsorption of p-nitrophenol present in aqueous solution has been demonstrated. As the pyrolysis temperature increased, the complex surface structure, larger surface area, higher aromaticity and lower hydrophobicity of the biochar were beneficial for p-nitrophenol adsorption. Notably, the aqueous solution’s pH and temperature also resulted in significant effects on p-nitrophenol adsorption. We also determined the reaction between p-nitrophenol and biochar to be spontaneous and endothermic, with a larger energy exchange taking place when biochar samples obtained at high pyrolysis temperature were utilized. Evidence indicated p-nitrophenol adsorption on biochar to be the result of both physical adsorption and chemisorption. In particular, the interactions between p-nitrophenol and biochar leading to the observed adsorption event comprised electrostatic interactions, H bonding, and π–π interactions. When biochar samples prepared performing the pyrolysis at high temperatures were utilized, the contribution to the adsorption of p-nitrophenol onto biochar of H bonding and π–π interactions was larger than when “low temperature” biochar samples were employed. The results of this study may provide theoretical guidance in the preparation of pine sawdust biochar and in the utilization of this product for the removal of p-nitrophenol from wastewater.

## Supplementary information

Supplementary Information.

## References

[1] Wu Z (2016). Enhanced adsorptive removal of p-nitrophenol from water by aluminum metal–organic framework/reduced graphene oxide composite. Sci. Rep..

[2] Xue G, Gao M, Gu Z, Luo Z, Hu Z (2013). The removal of p-nitrophenol from aqueous solutions by adsorption using gemini surfactants modified montmorillonites. Chem. Eng. J..

[3] Shen HM (2015). Fast adsorption of p-nitrophenol from aqueous solution using β-cyclodextrin grafted silica gel. Appl. Surf. Sci..

[4] Suja E, Nancharaiah YV, Venugopalan VP (2012). p-Nitrophenol biodegradation by aerobic microbial granules. Appl. Biochem. Biotechnol..

[5] Zhao P, Feng X, Huang D, Yang G, Astruc D (2015). Basic concepts and recent advances in nitrophenol reduction by gold- and other transition metal nanoparticles. Coord. Chem. Rev..

[6] Pourkhanali K (2018). Performance Evaluation of Bulk Liquid Membrane Technique on p-Nitrophenol Removal from Aqueous Solution. Chem. Biochem. Eng. Q..

[7] Zheng H (2017). Adsorption of p-nitrophenols (PNP) on microalgal biochar: Analysis of high adsorption capacity and mechanism. Bioresour. Technol..

[8] Ahmad M (2013). Trichloroethylene adsorption by pine needle biochars produced at various pyrolysis temperatures. Bioresour. Technol..

[9] Wang P (2017). Synthesis and application of iron and zinc doped biochar for removal of p-nitrophenol in wastewater and assessment of the influence of co-existed Pb(II). Appl. Surf. Sci..

[10] Chen X (2011). Adsorption of copper and zinc by biochars produced from pyrolysis of hardwood and corn straw in aqueous solution. Bioresour. Technol..

[11] Suliman W (2016). Modification of biochar surface by air oxidation: Role of pyrolysis temperature. Biomass Bioenergy..

[12] Choi YK, Kan E (2019). Effects of pyrolysis temperature on the physicochemical properties of alfalfa-derived biochar for the adsorption of bisphenol A and sulfamethoxazole in water. Chemosphere..

[13] Lonappan L (2016). Adsorption of methylene blue on biochar microparticles derived from different waste materials. Waste Manage..

[14] Essandoh M, Kunwar B, Pittman CU, Mohan D, Mlsna T (2015). Sorptive removal of salicylic acid and ibuprofen from aqueous solutions using pine wood fast pyrolysis biochar. Chem. Eng. J..

[15] Fomina M, Gadd GM (2014). Biosorption: current perspectives on concept, definition and application. Bioresour. Technol..

[16] He X (2018). Effects of pyrolysis temperature on the physicochemical properties of gas and biochar obtained from pyrolysis of crop residues. Energy..

[17] Chen YD, Lin YC, Ho SH, Zhou Y, Ren NQ (2018). Highly efficient adsorption of dyes by biochar derived from pigments-extracted macroalgae pyrolyzed at different temperature. Bioresour. Technol..

[18] Hameed BH, Ahmad AA, Aziz N (2007). Isotherms, kinetics and thermodynamics of acid dye adsorption on activated palm ash. Chem. Eng. J..

[19] Chen N (2010). Removal of fluoride from aqueous solution by adsorption onto kanuma mud. Water Sci. Technol..

[20] Jang HM, Yoo S, Choi YK, Park S, Kan E (2018). Adsorption isotherm, kinetic modeling and mechanism of tetracycline on pinus taeda-derived activated biochar. Bioresour. Technol..

[21] Zhou Y (2017). Modification of biochar derived from sawdust and its application in removal of tetracycline and copper from aqueous solution: Adsorption mechanism and modelling. Bioresour. Technol..

[22] Ding Z (2016). Sorption of lead and methylene blue onto hickory biochars from different pyrolysis temperatures: Importance of physicochemical properties. J. Ind. Eng. Chem..

[23] Zhang P, Li Y, Cao Y, Han L (2019). Characteristics of tetracycline adsorption by cow manure biochar prepared at different pyrolysis temperatures. Bioresour. Technol..

[24] Kambo HS, Dutta A (2015). A comparative review of biochar and hydrochar in terms of production physico-chemical properties and applications. Renew. Sustain. Energy Rev..

[25] Li S, Harris S, Anandhi A, Chen G (2019). Predicting biochar properties and functions based on feedstock and pyrolysis temperature: A review and data syntheses. J. Clean. Prod..

[26] Tan X (2015). Application of biochar for the removal of pollutants from aqueous solutions. Chemosphere..

[27] Li H (2017). Effect of pyrolysis temperature on characteristics and aromatic contaminants adsorption behavior of magnetic biochar derived from pyrolysis oil distillation residue. Bioresour. Technol..

[28] Angin D (2013). Effect of pyrolysis temperature and heating rate on biochar obtained from pyrolysis of safflower seed press cake. Bioresour. Technol..

[29] Akram M, Bhatti HN, Iqbal M, Noreen S, Sadaf S (2017). Biocomposite efficiency for Cr(VI) adsorption: Kinetic, equilibrium and thermodynamics studies. J. Environ. Chem. Eng..

[30] Tang D, Zheng Z, Lin K, Luan J, Zhang J (2007). Adsorption of p-nitrophenol from aqueous solutions onto activated carbon fiber. J. Hazard. Mater..

[31] Lazo-Cannata JC (2011). Adsorption of phenol and nitrophenols by carbon nanospheres: Effect of pH and ionic strength. Sep. Purif. Technol..

[32] Teng W (2018). Hierarchically porous carbon derived from metal-organic frameworks for separation of aromatic pollutants. Chem. Eng. J..

[33] Song J (2019). Highly efficient removal of Cr(VI) and Cu(II) by biochar derived from Artemisia argyi stem. Env. Sci. Pollut. Res..

[34] Sumalinog DAG, Capareda SC, de Luna MDG (2018). Evaluation of the effectiveness and mechanisms of acetaminophen and methylene blue dye adsorption on activated biochar derived from municipal solid wastes. J. Env. Manage..

[35] Phatai P, Klinkaewnarong J, Yaiyen S (2014). Adsorption of methyl violet dye from aqueous solutions by activated carbon produced from tamarind seeds. Adv. Mater. Res..

[36] Meng J (2014). Adsorption characteristics of Cu (II) from aqueous solution onto biochar derived from swine manure. Env. Sci. Pollut. Res..

[37] Ramadoss R, Subramaniam D (2018). Removal of divalent nickel from aqueous solution using blue-green marine algae: adsorption modeling and applicability of various isotherm models. Sep. Sci. Technol..

[38] Mousavi SJ, Parvini M, Ghorbani M (2018). Adsorption of heavy metals (Cu2+ and Zn2+) on novel bifunctional ordered mesoporous silica: optimization by response surface methodology. J. Taiwan. Inst. Chem. Eng..

[39] Zhang G (2011). Sorption of simazine to corn straw biochars prepared at different pyrolytic temperatures. Env. Pollut..

[40] Azargohar R, Nanda S, Kozinski JA, Dalai AK, Sutarto R (2014). Effects of temperature on the physicochemical characteristics of fast pyrolysis bio-chars derived from Canadian waste biomass. Fuel..

[41] Zhang B, Li F, Wu T, Sun D, Li Y (2015). Adsorption of p-nitrophenol from aqueous solutions using nanographite oxide. Colloids Surf. A: Physicochem. Eng. Asp..

[42] Bashir S, Zhu J, Fu Q, Hu H (2018). Comparing the adsorption mechanism of Cd by rice straw pristine and KOH-modified biochar. Env. Sci. Pollut. Res. Int..

