# Supplementary material for: Adsorption characteristics and mechanism of p-nitrophenol by pine sawdust biochar samples produced at different pyrolysis temperatures
Source: Sci Rep. 2020 Mar 20;10:5149. doi: 10.1038/s41598-020-62059-y (PMC7083892; doi:10.1038/s41598-020-62059-y)
Supplement: Supplementary file 1 — Supplementary Information. [file 41598_2020_62059_MOESM1_ESM.doc]

**Adsorption characteristics and mechanism of p-nitrophenol by** **pine sawdust biochar samples produced at different pyrolysis temperatures**

Lanqi Liu, Guozhi Deng, Xianyang Shi^*^

School of Resources and Environmental Engineering, Anhui University, Hefei, 230601, China

**^*^Corresponding Author:** Xianyang Shi

Fax: +86 551 63861970

1. mail: shixi381@163.com

**Supplementary data**

**Figure S1.** Effect of biochar (PC300) dosage on the adsorption of p-nitrophenol. Notably, in the adsorption experiments, the p-nitrophenol concentration in solution was 100 mg/L, the pH value was 6, the temperature was 35 °C, and the reaction time was 4 h. The reported data are the average of duplicate experiments, and the error bars indicate the standard error

**Figure. S2.** Effect of the solution's ionic strength on p-nitrophenol adsorption (q_e_: amount of p-nitrophenol adsorbed at equilibrium) by five biochar samples obtained at different pyrolysis temperatures (PC300, PC400, PC500, PC600, and PC700). Adsorption reaction conditions were as follows: 0.1 g of biochar was added to 20 mL of a 100 mg/L p-nitrophenol solution at pH 6 comprising a fixed concentration of NaCl (between 0.0 and 0.8 mol/L); the reaction was conducted under stirring at 35 °C for 4 h. The concentration of the p-nitrophenol left in the solution was then determined. The data reported are the average of duplicate experiments, and error bars indicate the standard error

**Figure S3.** Effect of environmental temperature on p-nitrophenol adsorption onto biochar samples obtained at different pyrolysis temperatures: (a) PC300, (b) PC400, (c) PC500, (d) PC600, and (e) PC700. In the adsorption experiments, 20 mL of a p-nitrophenol solution (50–800 mg/L, pH 6) and 0.1 g biochar were mixed together under stirring at three different temperatures (15 °C, 25 °C, and 35 °C) for a period of 48 h. The concentration of p-nitrophenol in the solution was then determined

**Figure S4.** FTIR of five biochar samples and the biochar loaded with p-nitrophenol (biochar after adsorbing p-nitrophenol) in the 400–4000 cm^−1^ wavenumber.
